# Beyond the First Year of Intravitreal Faricimab for Diabetic Macular Oedema: A Single-Centre Experience

**DOI:** 10.7759/cureus.89526

**Published:** 2025-08-07

**Authors:** Theodore Tsilegeridis-Legeris, Nihal Kenawy

**Affiliations:** 1 Ophthalmology, Liverpool University Hospitals NHS Foundation Trust, Liverpool, GBR

**Keywords:** anti-vegf treatment, diabetic macular edema (dme), diabetic macular oedema, faricimab, faricimab intravitreal injenction, intravitreal anti vegf, intravitreal injections, real-world, real-world data

## Abstract

Objective

To determine real-world clinical outcomes (including vision, anatomy and durability) of intravitreal faricimab (IVF) in year two (up to mean follow-up of 75 ± 15 weeks, range: 52-103 weeks) of treating diabetic macular oedema (DMO). Secondary objectives included assessing changes in diabetic retinopathy (DR) severity, the incidence of epiretinal proliferation (ERP)/epiretinal membrane (ERM), and safety.

Methodology

This is a single-centre retrospective observational study. Eligible eyes with ≥52 weeks follow-up, October 2022 to November 2024, were identified and categorised into naïve (no prior anti-vascular endothelial growth factor (anti-VEGF) agents) and switch (≥1 prior anti-VEGF). Descriptive statistics at week 52 (W52) and end of follow-up (EOFU) summarised mean BCVA and CFT change, mean IVF injections and frequency, DR severity change, epiretinal proliferation (ERP)/membrane (ERM) incidence, and safety.

Results

In total, 158 eyes (66 naïve, 92 switch) of 118 patients were identified, mean follow of 75 weeks. Mean W52 BCVA change was +3.6 (*P *= 0.04) for naïve and +1.3 letters (*P *= 0.34) for switch eyes; mean CFT reduction was -141 μm (*P *< 0.0001) and -115 μm (*P *< 0.0001), respectively. At EOFU, 27 (63%) naïve eyes and 36 (55%) switch eyes received IVF ≥12 weekly. In switch eyes, the mean W52 IVF interval was 11 weeks versus 7 with prior anti-VEGF agents (*P *< 0.0001). Of 115 available images, DR severity regressed or was stable in 111 (97%). ERP/ERM was developed in 10/39 (25.6%) naïve and 14/41 (34.1%) switch eyes. No safety outcomes were ever reported.

Conclusions

Faricimab demonstrated significant structural improvement and durability, with BCVA stability maintained into the second year of treatment. Most eyes showed regression or stability of DR with moderate ERP/ERM incidence.

## Introduction

Diabetic macular oedema (DMO) is the leading cause of vision loss in patients with diabetic retinopathy (DR). Over 18 million patients were affected globally in 2020, and over 28 million are predicted to be affected in 2045 [1]. Treatments inhibiting vascular endothelial growth factor (VEGF) are standard first-line therapy for DMO [2]; however, these require frequent hospital visits, leading to considerable treatment burden for patients, carers, and healthcare providers alike. Faricimab (Vabysmo/Genentech, Basel, Switzerland) was designed to inactivate VEGF-A as well as angiopoietin-2 as the first bispecific antibody for intraocular use, aiming to improve durability and clinical outcomes through dual pathway inhibition [3].

Outcomes after the first year of the YOSEMITE and RHINE phase III clinical trials demonstrated that intravitreal faricimab (IVF) improved visual acuity by 11.6 and 10.8 ETDRS letters, respectively, when administered using a personalised treat-and-extend (PTI) protocol with intervals of up to 16 weeks. In comparison, intravitreal aflibercept (Eylea/Regeneron, Tarrytown, NY) given at fixed eight-week intervals resulted in gains of 10.9 and 10.3 Early Treatment Diabetic Retinopathy Study (ETDRS) letters, respectively [3]. Non-inferior visual gains were also accompanied by favourable anatomical outcomes in the PTI arms of the YOSEMITE and RHINE trials (reduction in central subfield thickness of -196.5 and -187.6 μm, respectively) compared to eight weekly aflibercept (-170.3 and -170.1 μm, respectively) [3]. As a result, faricimab received U.S. Food and Drug Administration (FDA) approval for DMO in early 2022 and was approved in Great Britain in mid-2022 [4]. The subsequent long-term extension trial, RHONE-X, enrolled 1,223 patients who completed four years of total follow-up, of whom 415 were managed with faricimab according to a treat-and-extend regimen throughout [5]. Results from this study demonstrated that, at four years of follow-up, the visual and structural improvements achieved with faricimab were maintained, with treatment intervals extended up to 20 weeks and in the absence of DMO.

However, currently available real-world evidence on clinical outcomes of faricimab in treating DMO comprises preliminary data from larger, multicentre studies with up to a maximum of 12 months of follow-up, and smaller observational studies predominantly based on six months of follow-up or less [6], with only one case series describing outcomes at 12 months [7]. Data on longer-term outcomes are, therefore, lacking. In addition, to date, the effect of IVF on DR changes has not been reported, as it has been with aflibercept. Aflibercept is suggested to slow the progression of non-proliferative DR and may even reverse its severity [8-10]. Further, a post hoc analysis of YOSEMITE and RHINE data found IVF at eight weekly intervals reduced epiretinal membrane (ERM) formation over two years by 52% when compared to aflibercept given at the same frequency [11], but faricimab’s effect has not yet been studied in the context of everyday clinical practice.

This study primarily aims to assess the real-world clinical outcomes and treatment durability of intravitreal faricimab in patients with DMO during the second year of therapy. Second, it explores the impact of IVF on DR severity and ERM development in a clinical setting.

## Materials and methods

This study was registered with the Research, Development and Innovation Clinical Governance Department at St. Paul’s Eye Unit, Aintree University Hospital, Liverpool, United Kingdom (registration number: Ophth/CA/2024-25/04), and was conducted by the tenets of the Declaration of Helsinki [12] Adult participants (aged >18 years) who were being treated with IVF for DMO from October 2022 to November 2024 were identified from electronic patient records (©2024 Medisoft Limited) using an automated search function based on the study's inclusion criteria, and additional data were manually extracted. Eligible eyes in this study must have received at least four IVF injections, with the first administered at least 12 months before the study end date. Eyes were categorised into naïve (no prior anti-VEGF intravitreal injections) or switch (≥1 prior anti-VEGF intravitreal injection) for data analysis. Eyes were excluded from the study if they had received fewer than four IVF injections, regardless of whether they were naïve or switch eyes. At our treatment centre, eyes were treated with the treat and extend approach detailed in the YOSEMITE/RHINE clinical trials [3]. The only variation in approach was that some clinicians extended injection intervals by two weekly intervals rather than four weekly intervals. Switch eyes started faricimab based on departmental protocol for suboptimal response to other anti-VEGF agents.

Participants’ baseline data immediately before IVF initiation were collected, including age, sex, HbA1c closest to IVF initiation, as well as the number of prior anti-VEGF injections in the switch eyes. Parameters of best corrected visual acuity (BCVA), central foveal thickness (CFT; Heyex 2 ©2024 Heidelberg Engineering Inc.), DR grade on widefield imaging (Optos ®PLC), and epiretinal proliferation (ERP)/epiretinal membrane (ERM) status [13,14] (Heyex 2) were also collated from before IVF initiation, at week 52 (W52), and end of follow-up (EOFU). Participant BCVA was primarily recorded in patient records using ETDRS letters. On rare occasions when logMAR or Snellen scales were used, these values were converted to ETDRS letters using established methods [15,16]. Primary effectiveness outcomes were mean change in BCVA and CFT at W52 and EOFU, compared to baseline (immediately before IVF initiation). Durability outcomes were the mean number of IVF injections given in the first vs. second 6 months of treatment, and their frequency at W52 and EOFU. In switch eyes, IVF interval at W52 and EOFU was compared to those with other anti-VEGF agents before switching to faricimab. Suboptimal response to treatment was defined as a visual gain of <5 letters and/or <20% reduction in CFT [17-20]. Eyes exhibiting suboptimal response were considered for commencing alternative treatment after discussion with the patient.

Secondary outcomes were the DR severity change on widefield imaging and ERP/ERM incidence. Images were all assessed by the senior author and retina specialist (NK). Grade of DR was categorised as follows: R1 - background DR, R2 - pre-proliferative DR, R3A - active proliferative DR, and R3S - stable proliferative DR [21]. Tables 1-2 define ERP, ERM, and the DR severity grades used in this study. Lastly, records were also searched for safety endpoints of intraocular inflammation, endophthalmitis, vasculitis, and retinal artery occlusion.

**Table 1:** Definitions of epiretinal proliferation (ERP) and epiretinal membrane (ERM) used when reviewing OCT images in this study. The stage of ERM was not used for data analysis; only the presence or absence of ERP/ERM was considered. OCT, optical coherence tomography

Pathology		Features
ERP		Iso-reflective space-filling material over the retinal surface, often delimited by a thin, highly reflective line, and without tractional properties [13].
ERM	Stage 1	Mild and thin ERM, with the foveal depression present. All retinal layers are clearly defined on OCT.
	Stage 2	ERM is associated with widening of the outer nuclear layer and loss of the foveal depression. All retinal layers are clearly defined on OCT.
	Stage 3	ERM is associated with continuous ectopic inner foveal layers crossing the entire foveal area. All retinal layers are clearly defined on OCT.
	Stage 4	Thick ERM associated with continuous ectopic inner foveal layers [14].

**Table 2:** Definitions of DR severity grades used in this study when reviewing fundus images.

Grade	Description	Features
R1	Background	Microaneurysms; retinal haemorrhages; venous loops; cotton wool spots in the presence of other R1 features; retinal exudates ± exudates.
R2	Pre-proliferative	Venous beading and reduplications; intraretinal microvascular abnormality visible on colour photography; multiple deep, round or blot haemorrhages.
R3A	Active proliferative	New vessels on the disc; new vessels elsewhere; pre-retinal or vitreous haemorrhage; pre-retinal fibrosis ± tractional retinal detachment.
R3S	Stable proliferative	Treated with panretinal photocoagulation and stable.

Statistical analyses were performed on Microsoft Excel (©2024 Microsoft Excel Version 16.88). Data were categorised into parametric and non-parametric distributions using the Kolmogorov-Smirnov test. Parametric and non-parametric data were analysed using the paired samples t-test and the Wilcoxon Signed Rank test, respectively. Where applicable, results are presented as the mean value ± standard deviation (median value).

## Results

In total, 158 eyes of 118 patients (66 naïve and 92 switch) met the study inclusion criteria; baseline characteristics are provided in Table 3. Mean follow-up was 75 weeks ± 15 weeks, with 26 naïve (39.4%) and 40 switch eyes (43.5%) being followed up for >18 months. After four monthly loading injections at initiation of IVF, naïve eyes were managed with the treat-and-extend approach mentioned in our methods. Switch eyes were loaded with IVF at intervals according to their pre-switch injection frequency and then managed with the treat-and-extend approach previously mentioned. Intervals in the real world were extended up to 16-week intervals, as in the clinical trials. In two eyes, clinicians chose to stop injections and monitor with future injections on a pro re nata basis if vision and CFT remained stable at 16 weekly intervals. There was, unfortunately, no specific monitoring schedule for this; however, these eyes were removed from data analysis for durability.

**Table 3:** Baseline characteristics of participants before treatment with faricimab. Anti-VEGF, anti-vascular endothelial growth factor; BCVA, best-corrected visual acuity; CFT, central foveal thickness; DR, diabetic retinopathy; ERP, epiretinal proliferation; ETDRS, Early Treatment Diabetic Retinopathy Study; HbA1c, glycated haemoglobin; IVF, intravitreal faricimab; SD, standard deviation

Baseline characteristic		Whole cohort	Naïve	Switch
Number of eyes		158	66	92
Number of patients		118	50	68
Patient age, mean years (SD)		64 (12)	62 (12)	65 (13)
Patient sex	Female, *n* (%)	50 (42%)	28 (56%)	22 (32%)
	Male, *n* (%)	68 (58%)	22 (44%)	46 (68%)
HbA1c before IVF initiation, mean mmol/mol (SD)		69.4 (16.2)	70.1 (16.6)	68.9 (16.1)
Anti-VEGF injections before IVF, mean (SD)		-	-	11.3 (8.2)
Mean BCVA, ETDRS letters (SD)		60.7 (15.0)	64.0 (12.4)	58.3 (16.3)
Mean CFT, μm (SD)		434 (118)	466 (97)	410 (126)
Eyes with ERP , *n* (%)		72 (46%)	25 (38%)	47 (52%)
DR grade, *n* eyes (%)				
-R1: background DR		59 (37%)	22 (33%)	37 (40%)
-R2: pre-proliferative DR		42 (27%)	24 (36%)	18 (20%)
-R3S: stable proliferative DR		42 (27%)	13 (20%)	29 (32%)
-R3A: active proliferative DR		15 (9%)	7 (11%)	8 (9%)

There were 149 eyes at W52 (63 naïve and 86 switch) and 144 eyes at EOFU (61 naïve and 83 switch) that continued to receive or had successfully completed IVF treatment and were undergoing monitoring only (Table 4). Nine eyes were excluded from W52 analyses and a further five were excluded from EOFU analyses because of suboptimal response to IVF for which alternative treatments were commenced. Data on the 14 excluded eyes are presented in Table 5.

**Table 4:** Number of eyes included in data collection and analysis before IVF initiation (baseline), at week 52 (W52) and at end of follow-up (EOFU). The cumulative mean number of IVF injections given at each point in time are also presented. EOFU, end of follow-up; IVF, intravitreal faricimab; SD, standard deviation; W52, week 52

	Naïve		Switch	
	Eyes included in study, *n* (%)	Cumulative number of IVF injections given, mean ± SD	Eyes included in study, *n* (%)	Cumulative number of IVF injections given, mean ± SD
Baseline	66	0	92	0
W52	63	6.9 ± 1.5	86	7.5 ± 1.6
EOFU	61	8.5 ± 2.1	83	9.0 ± 2.5

**Table 5:** Eyes that stopped IVF and started other treatment during follow-up were excluded from data analysis. Change in BCVA and CFT are all from baseline (before faricimab). Anti-VEGF, anti-vascular endothelial growth factor; BCVA, best-corrected visual acuity; CFT, central foveal thickness; DR, diabetic retinopathy; EOFU, end of follow-up; ETDRS, Early Treatment Diabetic Retinopathy Study; IVF, intravitreal faricimab; N, naïve; S, switch; W52, week 52

Eye		DR grade before IVF	No. IVF doses	Excluded at	BCVA change when IVF stopped, ETDRS letters	CFT change when IVF stopped, μm	DR grade W52	Treatment commenced after stopping IVF	Change in BCVA EOFU, ETDRS letters	Change in CFT EOFU, μm	DR grade EOFU
N	1	R2	5	W52 + EOFU	-15	-118	R1	Ozurdex	-23	12	R2
	2	R1	6	W52 + EOFU	-20	-29	R1	Eylea, then Ozurdex	-1	-94	R1
	3	R3S	6	EOFU	-10	-34	R3S	Ozurdex	-16	39	R3S
	4	R3A	5	W52 + EOFU	2	-43	R3S	Ozurdex	4	-82	R3A
	5	R3S	9	EOFU	6	8	R3S	Ozurdex	3	94	R3S
S	6	R3S	5	W52 + EOFU	15	-419	R3S	Ozurdex	6	-399	R3A
	7	R1	4	W52 + EOFU	-8	32	R1	Ozurdex	3	39	R1
	8	R3S	11	EOFU	0	-104	R3S	Macular laser – grid, then Avastin	1	-94	R3A
	9	R3A	5	W52 + EOFU	-1	-79	R3S	Avastin, then macular laser – grid	-16	-101	R3S
	10	R3S	6	W52 + EOFU	-6	-28	R3S	Ozurdex	3	-208	R3S
	11	R1	6	W52 + EOFU	-32	18	R1	Ozurdex	-36	45	R1
	12	R1	7	EOFU	-31	Not assessable due to cataract	R1	Cataract surgery, then Ozurdex	19	-83	R1
	13	R2	8	EOFU	0	-80	R1	Ozurdex	0	-108	R1
	14	R3A	10	W52 + EOFU	-27	149	R3S	Ozurdex, then Avastin	-38	229	R3S

Primary outcomes: vision, anatomy and durability 

**Figure 1:**
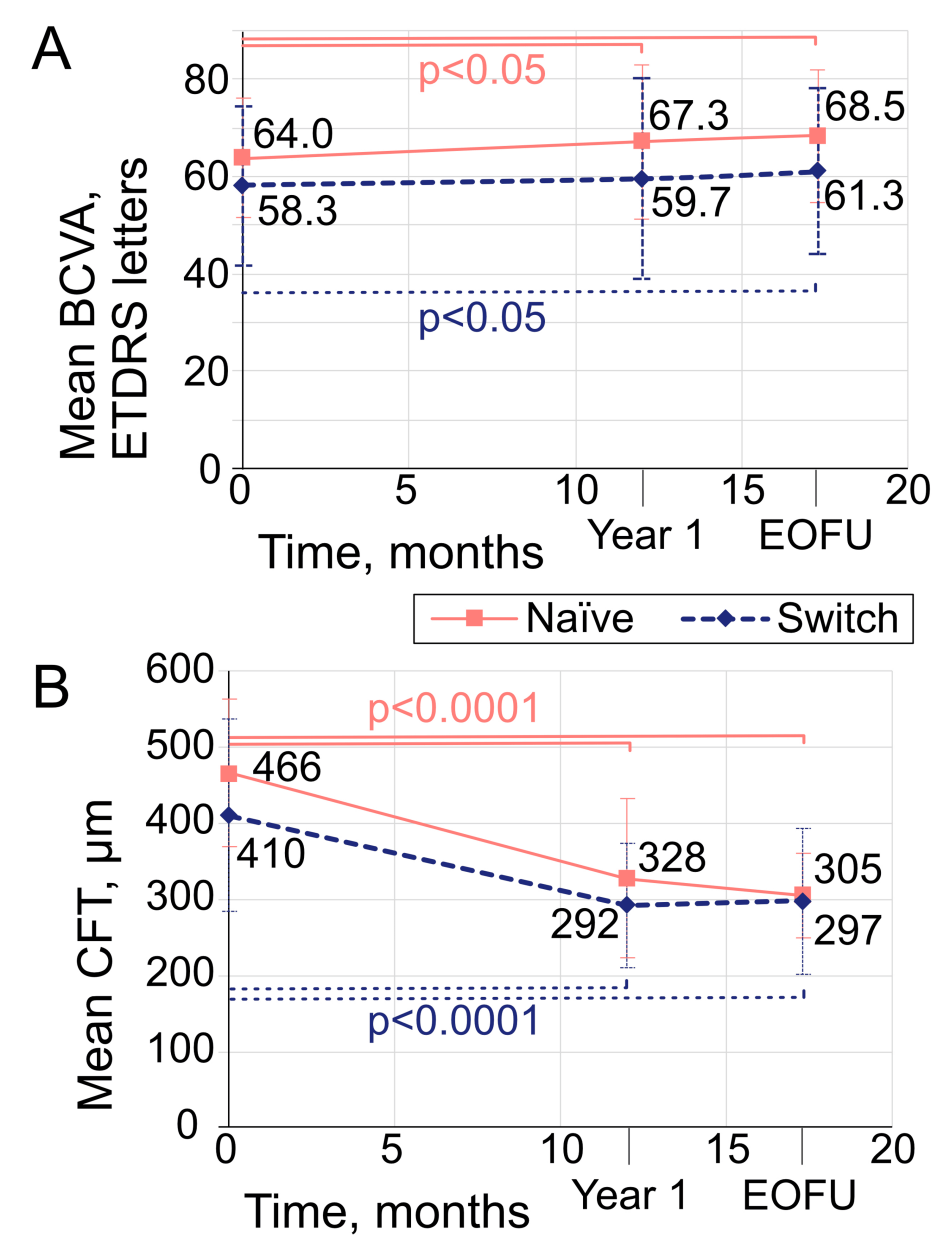
Mean BCVA (A) and CFT (B) in naïve and switch eyes at baseline, W52 and EOFU. Error bars represent standard deviation. The differences between means here vary slightly from the manuscript text because baseline, W52 and EOFU means here were calculated from all 158, 149 and 144 eyes, respectively, whereas results in the study do not include baseline data for the eyes excluded from data analyses (Table 3). BCVA, best-corrected visual acuity; CFT, central foveal thickness; EOFU, end of follow-up; ETDRS, Early Treatment Diabetic Retinopathy Study; W52, week 52

From all BCVA measurements across the study, 8.3% used logMAR or Snellen acuity, which were converted to ETDRS letters, as outlined in our methods, to facilitate data analysis. All remaining BCVA measurements were taken using ETDRS charts and recorded in ETDRS letters. At W52, for the 149 analysed eyes, BCVA mean change from baseline was +3.6 ± 13.4 ETDRS letters (median: +3.8); *P *= 0.039 for naïve eyes and +1.3 ± 12.4 letters (median: +2.0); *P *= 0.335 for switch eyes (Figure 1). At EOFU compared to baseline, mean change in BCVA in naïve eyes was +3.6 ± 11.9 ETDRS letters (median: +3.0); *P *= 0.039, and +1.6 ± 14.6 letters (median: +2.9); *P *= 0.032 in switch eyes (Figure 1). 

As for CFT, at W52, mean change from baseline was -141 μm ± 127 μm (median: -157 μm); *P *< 0.0001 for naïve eyes, and -115 μm ± 133 μm (median: -95); *P *< 0.0001 for switch eyes (Figure 1). At EOFU, mean change in CFT in naïve eyes was -156 μm ± 111 μm (median: -159); *P *< 0.0001 (Figure 1). In switch eyes, mean change in CFT at EOFU was -109 μm ± 130 μm (median: -101); *P *< 0.0001 (Figure 1).

By EOFU, of the 144 eyes included, naïve eyes received 8.5 ± 2.1 mean injections, whereas switch eyes received 9.0 ± 2.5 mean injections (Table 4). Before initiating IVF, these switch eyes had received 11.3 ± 8.2 injections of other anti-VEGF agents at a mean interval of 7.3 ± 4.6 weeks between the last two injections. Among the 83 switch eyes included in EOFU analysis, 52 eyes (62.7%) had experienced one type of anti-VEGF agent, 27 (32.5%) had experienced two distinct agents and 4 (4.8%) had experienced three distinct anti-VEGF agents (Table 6). Aflibercept was the latest anti-VEGF agent received by 56 eyes (67.5%) before IVF initiation. Bevacizumab was the latest agent in 17 eyes (20.5%) and ranibizumab in 10 eyes (12.0%).

**Table 6:** Number of distinct anti-VEGF agents received by switch eyes before IVF initiation, and the anti-VEGF agent last administered before IVF initiation. Data are presented for the 83 switch eyes that were followed up until the end of the study only. Anti-VEGF, anti-vascular endothelial growth factor

Characteristic	Eyes, *n* (%)	Characteristic	Eyes, *n* (%)
1 anti-VEGF agent pre-switch	52 (62.7%)	Aflibercept pre-switch	56 (67.5%)
2 anti-VEGF agents pre-switch	27 (32.5%)	Bevacizumab pre-switch	17 (20.5%)
3 anti-VEGF agents pre-switch	4 (4.8%)	Ranibizumab pre-switch	10 (12.0%)

**Figure 2:**
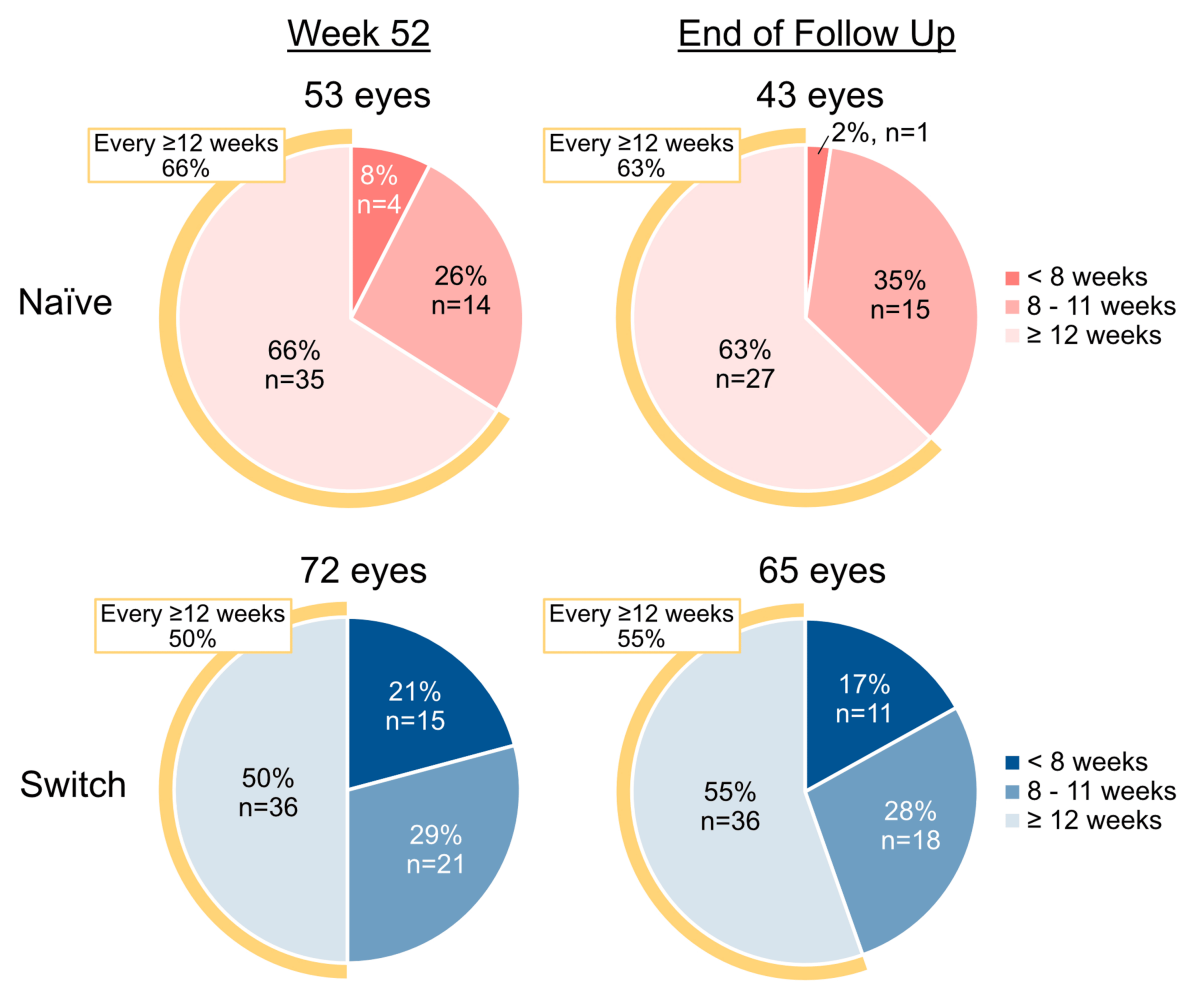
Intervals between IVF injections at W52 and EOFU in naïve and switch eyes. IVF, intravitreal faricimab; EOFU, end of follow-up

During the first year of treatment, the mean number of IVF injections in the first versus second 6 months was 4.7 vs. 2.2 in naïve eyes (*P* < 0.0001), and 4.8 vs. 2.7 in switch eyes (*P* < 0.0001). Of the 66 eyes followed up for more than 18 months, 17 naïve eyes and 27 switch eyes continued to receive IVF, with a mean of 2.3 and 2.8 injections, respectively, during their third six-month treatment period. At W52, 53/63 naïve (84.1%) and 72/86 switch eyes (83.7%) were receiving regular IVF. The mean interval between IVF injections was 12.9 ± 4.6 weeks in naïve eyes and 11.5 ± 4.0 weeks in switch eyes. More specifically, 35/53 naïve (66.0%) and 36/72 switch eyes (50.0%) received IVF at ≥12 weekly intervals (Figure 2).

At EOFU, 43/61 naïve (70.5%) and 65/83 switch eyes (78.3%) were receiving ongoing IVF. The mean intervals between injections were 12.9 ± 4.4 weeks in naïve eyes and 11.5 ± 4.0 weeks in switch eyes. Of these, 27/43 naïve (62.8%) and 36/65 switch eyes (55.4%) received IVF at ≥12 weekly intervals (Figure 2).

Secondary outcomes: DR severity and ERP/ERM

**Figure 3:**
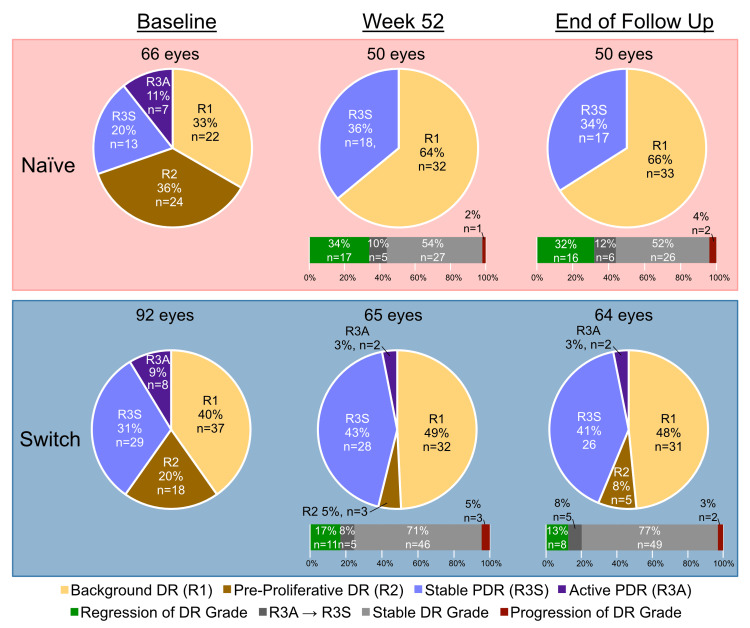
DR severity at baseline, W52 and EOFU in naïve and switch eyes. DR, diabetic retinopathy; PDR, proliferative diabetic retinopathy; EOFU, end of follow-up

With regards to DR severity, at W52, Optos images were available for 115/149 eyes (77.2%) across the whole cohort. In total, DR severity regressed in 28/115 eyes (24.3%) from R2 to R1, DR grade was stable in 83/115 eyes (72.2%) and progressed in 4/115 eyes (3.5%); one from R1 to R2, one from R1 to R3A and two from R2 to R3A (Figure 3). At EOFU, Optos images were available for 114/144 eyes (79.2%); DR severity regressed in 24/114 eyes (21.1%) from R2 to R1, DR grade was stable in 86/114 eyes (75.4%) and progressed in one further eye from R1 to R2, meaning 4/114 eyes (3.5%) showed progression of DR during the entirety of follow up (Figure 3).

As for ERP/ERM, at baseline, there were 157 assessable OCTs from the 158 study eyes, of which there were 85 eyes - 41/66 (62.1%) naïve and 44/91 (48.4%) switch eyes - that did not have evidence of ERP/ERM. By W52, 5 eyes were excluded due to suboptimal IVF response and OCTs available for 71 remaining eyes showed 8/36 naïve (22.2%) and 9/35 switch eyes (25.7%) had developed ERP/ERM, and 63 eyes across the whole cohort remained with no evidence of ERP/ERM. At EOFU, OCTs were available for 61 of the 63 (96.8%) eyes, showing 2/30 (6.7%) naïve and 5/31 (16.1%) switch eyes had developed ERP/ERM. Therefore, over the entirety of mean 75 weeks follow up, 10/39 (25.6%) naïve and 14/41 (34.1%) switch eyes developed ERP/ERM after the initiation of IVF therapy.

No safety outcomes were ever reported during follow up.

## Discussion

This study reports data with a mean follow-up of 75 weeks on the use of faricimab in treating DMO at a single centre in the United Kingdom. To our knowledge, this is the longest currently available follow-up of real-world outcomes of IVF in DMO management. 

In terms of BCVA, in our cohort, there was a minimal gain of +3.6 letters from a baseline of 64.0 ETDRS letters in naïve eyes, denoting stability of vision. However, this visual improvement was less than the 6.2 ETDRS letter gain from a baseline of 63.7 letters reported at 12 months in 176 treatment-naïve eyes in the UK-based FAREWIDE-DMO study [22]. In comparison, our treatment-experienced eyes achieved +1.3 ETDRS letter gain from baseline mean BCVA of 58.3 letters; this is similar to FAREWIDE-DMO +1.0 letter improvement in 489 previously treated eyes, albeit from a higher baseline BCVA of 67.3 letters [22]. The method of BCVA assessment was unlikely to have affected our final results because only a small proportion used Snellen or logMAR acuity, and these were converted to ETDRS letters using internationally recognised conversion methods [15,16].

In addition, IVF demonstrated good durability in our cohort with a mean of 6.9 injections in naïve eyes and 7.5 injections in switch eyes during the first year of treatment, with a further 2.3 mean injections in naïve eyes and 2.8 mean injections in switch eyes during the third six months of treatment. Injections were marginally more frequent in the first year of treatment than the 6.1 and 7.0 injections in the FAREWIDE cohort [22]. One reason for slightly more frequent IVF injections in our cohort could be the cautious extensions of injection intervals by 2 weekly increments, in pursuit of optimising personalised extension strategies rather than the recommended 4 weekly extensions described in the clinical trials [3]. The slightly smaller visual improvement in our population of naïve eyes despite slightly more IVF injections compared to FARWIDE could be the result of our smaller cohort size and, perhaps, discrepancy in study population characteristics, such as race, ethnicity, age, glycaemic control, and duration of diabetic eye disease, impacting ocular tissue health. Nonetheless, both our study and FARWIDE-DMO have shown a smaller BCVA change compared to the YOSEMITE and RHINE clinical trials [3], in which, after one year of IVF in the personalised treat-and-extend (PTI) arms (*n* = 313 for YOSEMITE and *n* = 319 for RHINE, comprising 78% and 80% anti-VEGF naïve eyes, respectively), there was BCVA gain of +11.6 (mean baseline 61.9 ETDRS letters) and +10.8 (mean baseline 62.5 ETDRS letters), respectively [3]. Our report and the FARWIDE-DMO data are reflections of real-world clinical practice rather than the stringent criteria chosen by the pivotal trials.

In comparison to existing observational literature, our study is the first to provide detailed results of IVF in both treatment-naïve and anti-VEGF-experienced (switch) eyes beyond the first 12 months of treatment. However, real-world clinical evidence is significantly lagging behind that of the RHONE-X four-year extension trial [5]. The ongoing, multicentre FARWIDE [22] and FARETINA [23] studies collecting real-world data are summarised in Table 7 alongside data from RHONE-X [5].

**Table 7:** Summary of ongoing large multicentre real-world studies reporting outcomes of faricimab in treating DMO. N/A, not applicable; NR, not reported; DMO, diabetic macular oedema *Median value.

Study	Country	Study population	Eyes	Follow-up	No. of IVF injections	Mean change in BCVA, ETDRS letters	Mean change in CFT or equivalent, μm	Mean IVF interval at EOFU
FARWIDE [22]	United Kingdom	Naïve	215	12 months	6.1	5.5	NR	NR
		Switch	579	12 months	7.0	0.9	NR	NR
FARETINA [23]	United States	Naïve	538	12 months	5.3	NR	NR	NR
		Switch	3683	12 months	6.6	NR	NR	NR
RHONE-X [5]	International	Naïve and switch eyes from YOSEMITE/RHINE clinical trials (T&E arm)	506	4 years	18*	10.1	-198.3	NR

Our study found that 56.8% of all eyes, whether naïve or anti-VEGF experienced, achieved ≥12 weekly injection intervals at W52, and 58.3% of all eyes were maintained at ≥12 weekly intervals at 75 weeks mean follow-up. Moreover, in switch eyes, the IVF injection intervals achieved at W52 and thereafter were, on average, 4 weeks longer than with any other anti-VEGF agent before switching, despite switch eyes having received an average of 11.9 prior anti-VEGF injections. Our observation of longer treatment intervals with IVF is consistent with previously published literature [24-26]. Ohara et al. [25] reported outcomes of 18 eyes with DMO refractory to ranibizumab or aflibercept that were switched to IVF on a PRN basis. Their results showed that after a mean follow-up of 6.1 months, during which 2.8 mean IVF injections were administered, IVF injection intervals were on average five weeks longer than with the other two agents, but with no statistically significant BCVA or central foveal thickness (CFT) improvement. Similarly, Durrani et al. reported that 36.2% (*n* = 25) of their cohort of 69 eyes with DMO resistant to aflibercept, ranibizumab, and/or ranibizumab were able to be extended by ≥ 2 weeks after an average of three months of IVF treatment [24]. This occurred with a statistically significant reduction in mean CFT (*P *< 0.001) and stable visual acuity. Finally, Borchert et al. [26] found that intervals between IVF injections were on average 1.4 weeks longer (*P *< 0.05) after six months and 5.7 mean IVF injections than in 44 eyes with partial response to aflibercept.

Besides BCVA and CFT, we were uniquely able to evaluate the effect of IVF on DR severity and ERP/ERM development in a real-world context, which, to our knowledge, has not been done previously. In our cohort, there was DR grade improvement in 32% of naïve eyes and 13% of switch eyes, and stability in 64% of naïve eyes and 85% of switch eyes during the 75-week mean follow-up, further supporting a stabilising effect of the angiopoietin-2 component of faricimab in DR. However, these findings should be interpreted with caution. It is not clear if IVF alone was a contributing factor to such regression/ stabilisation or whether improved glycaemic and systemic risk factors also played a role. Further longitudinal and larger studies with assessment of systemic confounders would help shed light on our observation.

With regards to ERP/ERM, our results showed that the pooled incidence of ERP/ERM among both naïve and switch eyes at EOFU was 30% (24/80). This percentage is higher than the two-year post hoc analysis of pooled YOSEMITE and RHINE clinical trial data, which found a lower incidence of ERM in the eight-weekly faricimab arm of 619 eyes (3.8%) compared to the eight-weekly aflibercept arm of 604 eyes (7.6%) when followed up until week 100 [11]. There was no statistically significant difference in ERM between the faricimab PTI arm and eight-weekly aflibercept at week 100 [11]. It is of note that comparing the effect of aflibercept to IVF on ERP/ERM formation was not within the scope of our study; however, faricimab’s benefit over aflibercept in this respect may be slight, requiring a large number of injections over a prolonged period to produce an observable benefit. Notably, the YOSEMITE/RHINE post-hoc analysis defined ERM as the presence of a membrane on the ILM causing significant distortion of macular architecture in the central subfield [11], whereas we examined both ERP, as a potential precursor, and all ERM stages to capture all changes that could be linked to IVF. This likely explains the higher incidence in our cohort alongside other confounding factors, such as differing inclusion and exclusion criteria, and population factors, e.g., duration of DR, glycaemic control, and HbA1c levels, which were higher in our study than in the clinical trials [3].

In the current study, there was a suboptimal response to faricimab in 14 eyes (5 naïve and 9 switch), and other treatments for DMO had to be initiated (Table 5). Ozurdex was given in 12 of these cases, and the remaining two cases were treated with a different anti-VEGF agent and macular laser. Outcomes at the end of follow-up in these 14 eyes were largely similar to or worse than outcomes with faricimab. Possible reasons for suboptimal response to IVF include a delay in the initiation of anti-VEGF therapy [20], which may have been exacerbated by the requirement for patients to have a CFT > 400 μm before starting treatment in England, as per NICE guidance at the time of this study [4]. Other possible explanations for suboptimal response include duration of DR [27], poorer glycaemic control, and the presence of macular ischaemia [28]. We did not specifically look for macular ischaemia because of the limited availability of fundus fluoroscein angiography (FFA) and OCT angiography (OCTA) imaging. Such imaging was not routinely performed in baseline assessment of DMO and would only be requested following a lack of response to anti-VEGF treatment. Notwithstanding, the availability of FFA would be influenced by contraindications such as severe kidney disease, needle phobia, and allergy, whereas OCTA was not available at our treatment centre until mid-2024.

The strengths of our study include its broad inclusion criteria, which reflect real-world patients and outcomes as closely as possible, as well as the larger sample size and longer follow-up than preceding observational studies. Furthermore, we have evaluated naïve and switch cohorts separately to improve the generalisability of results and explored novel areas of faricimab’s effect on DR severity and ERP/ERM incidence in a real-world context.

However, we acknowledge the key limitations of our single-centre study’s observational design without control participants. The single-centre nature of our study limits the generalisability of our results. Variability in decision-making by clinicians regarding extension intervals and factors that affect the planning of injections or monitoring based on capacity and demand will likely vary in other locations. The observational nature of our study without a control group receiving a different treatment means causality cannot be established, and faricimab cannot be definitively compared to other treatments. There are potential confounding factors, such as changes in HbA1c during treatment or DR severity affecting ERP/ERM formation [29], that we have not accounted for, and more detailed data or randomisation with a control group would improve the validity of our results in this regard. Our results, particularly regarding our secondary outcomes, must therefore be interpreted with caution. Despite adopting the treat and extend protocol used in the YOSEMITE/RHINE clinical trials [3], there was some variability in treatment approach, with a very small proportion of eyes being monitored for pro re nata treatment if CFT and BCVA were stable at 16 weekly injections. As these eyes were removed from data analysis for durability and are small in number, they are unlikely to impact our final results. The lack of masking and the lack of multiple graders when reviewing OCT scans and fundus images for DR grades and ERP/ERM incidence are further limitations that introduce a risk of bias and limit the replicability of our results. The availability of data in our study is influenced by real-world capacity in outpatient clinics, and so data may be missing or approximated at W52 and EOFU timepoints, or data may be missing for other reasons, such as loss to follow-up. Unfortunately, missing data can introduce a risk of bias and reduce the power of the study. Lastly, we acknowledge that a dedicated software package would have been better suited for data analysis, although the software used was functional, and we acknowledge the inherent limitations of our assessment of safety that may have missed transient or mild events.

## Conclusions

In summary, from our clinical experience, faricimab demonstrated good durability at one year and into year two, allowing ≥12 weekly injection intervals in the majority of eyes, while maintaining vision and improving CFT in naïve and switch eyes alike. In eyes previously treated with other anti-VEGF agents and unable to extend beyond 8-10 weekly injections, faricimab increased the injection interval by an average of four weeks. However, real-world visual acuity improvements are not as significant as clinical trial data suggest, which may be due to cohort characteristics and less stringent inclusion criteria in our study.

Faricimab may possibly have a role in slowing the progression of DR and limiting ERP/ERM development; however further studies, with control participants, are required to evaluate our observations and investigate this further.

## References

[1] Teo ZL, Tham YC, Yu M (2021). Global prevalence of diabetic retinopathy and projection of burden through 2045: systematic review and meta-analysis. Ophthalmology.

[2] (2025). Diabetic retinopathy: management and monitoring (NG242). Diabetic Retinopathy.

[3] Wykoff CC, Abreu F, Adamis AP (2022). Efficacy, durability, and safety of intravitreal faricimab with extended dosing up to every 16 weeks in patients with diabetic macular oedema (YOSEMITE and RHINE): two randomised, double-masked, phase 3 trials. Lancet.

[4] (2025). Faricimab for treating diabetic macular oedema (TA799). Faricimab for treating diabetic macular oedema.

[5] Khanani AM, Abreu F, Gibson K (2025). Four-Year Outcomes of Faricimab in DME: Safety and Efficacy Results From The RHONE-X Long-Term Extension Trial; Presented at the Macula Society 48th Annual Meeting; Charlotte Harbour, FL, 12-15 February 2025. https://medically.gene.com/global/en/unrestricted/ophthalmology/MACULA-SOCIETY-2025/macula-society-2025-presentation-khanani-four-year-outc.html?cid=slprxx2106ongaoncology-annual-meeting2021.

[6] Nasimi S, Nasimi N, Grauslund J, Vergmann AS, Subhi Y (2024). Real-world efficacy of intravitreal faricimab for diabetic macular edema: a systematic review. J Pers Med.

[7] Rush RB (2023). One year results of faricimab for aflibercept-resistant diabetic macular edema. Clin Ophthalmol.

[8] Maturi RK, Glassman AR, Josic K (2021). Effect of intravitreous anti-vascular endothelial growth factor vs sham treatment for prevention of vision-threatening complications of diabetic retinopathy: the Protocol W randomized clinical trial. JAMA Ophthalmol.

[9] Brown DM, Wykoff CC, Boyer D (2021). Evaluation of intravitreal aflibercept for the treatment of severe nonproliferative diabetic retinopathy: results from the PANORAMA randomized clinical trial. JAMA Ophthalmol.

[10] Simmonds M, Llewellyn A, Walker R (2024). Anti-VEGF drugs compared with laser photocoagulation for the treatment of diabetic retinopathy: a systematic review and meta-analysis. Health Technol Assess.

[11] Jaffe GJ, Deák G, Gibson K (2025). Impact of faricimab versus aflibercept on epiretinal membrane formation over 2 years in patients with diabetic macular edema in the phase 3 YOSEMITE and RHINE trials. Retina.

[12] (2013). World Medical Association Declaration of Helsinki: ethical principles for medical research involving human subjects. JAMA.

[13] Chehaibou I, Pettenkofer M, Govetto A, Rabina G, Sadda SR, Hubschman JP (2020). Identification of epiretinal proliferation in various retinal diseases and vitreoretinal interface disorders. Int J Retina Vitreous.

[14] Hung CL, Lin KH, Lee YK, Mrozek D, Tsai YT, Lin CH (2023). The classification of stages of epiretinal membrane using convolutional neural network on optical coherence tomography image. Methods.

[15] Beck RW, Moke PS, Turpin AH (2003). A computerized method of visual acuity testing: Adaptation of the early treatment of diabetic retinopathy study testing protocol. Am J Ophthalmol.

[16] Gregori NZ, Feuer W, Rosenfeld PJ (2010). Novel method for analyzing snellen visual acuity measurements. Retina.

[17] Dugel PU, Campbell JH, Kiss S (2019). Asociation between eary anatomic response to anti-vascular endothelial growth factor therapy and long-term outcome in diabetc macular edema. Retina.

[18] Gonzalez VH, Campbell J, Holekamp NM (2016). Early and long-term responses to anti-vascular endothelial growth factor therapy in diabetic macular edema: analysis of Protocol I data. Am J Ophthalmol.

[19] Pinto M, Mathis T, Massin P (2021). Visual acuity gain profiles and anatomical prognosis factors in patients with drug-naive diabetic macular edema treated with dexamethasone implant: the NAVEDEX study. Pharmaceutics.

[20] Rennie C, Lotery A, Payne J, Singh M, Ghanchi F (2024). Suboptimal outcomes and treatment burden of anti-vascular endothelial growth factor treatment for diabetic macular oedema in phakic patients. Eye (Lond).

[21] (2025). NHS England. NHS Diabetic Eye Screening Programme: grading definitions for referable disease.. https://www.gov.uk/government/publications/diabetic-eye-screening-retinal-image-grading-criteria/nhs-diabetic-eye-screening-programme-grading-definitions-for-referable-disease-start-date-october-01.

[22] Kiire C, Reynolds R, Peto T (2024). Real-World Treatment Patterns and Visual Outcomes in the First 12 Months of Faricimab Use Among Eyes With DMO in the UK: Results From the FARWIDE-DMO Study. Oxford Ophthalmological Congress 2024 Oxford, UK.

[23] Leng T, Borkar D, Tabano D (2025). 12-Month Real-World Clinical and Anatomical Outcomes With Faricimab in Patients With Diabetic Macular Edema: The FARETINA-DME Study; Presented at the American Society of Retina Specialists 2024 Meeting; Stockholm, Sweden, 17-20 July 2024. https://medically.gene.com/global/en/unrestricted/ophthalmology/ASRS-2024/asrs-2024-presentation-leng-real-world-clinical-and-ana.html.

[24] Durrani AF, Momenaei B, Wakabayashi T (2024). Conversion to faricimab after prior anti-vascular endothelial growth factor therapy for persistent diabetic macular oedema. Br J Ophthalmol.

[25] Ohara H, Harada Y, Hiyama T, Sadahide A, Minamoto A, Kiuchi Y (2023). Faricimab for diabetic macular edema in patients refractory to ranibizumab or aflibercept. Medicina (Kaunas).

[26] Borchert GA, Kiire CA, Stone NM (2024). Real-world six-month outcomes in patients switched to faricimab following partial response to anti-VEGF therapy for neovascular age-related macular degeneration and diabetic macular oedema. Eye (Lond).

[27] Gurung RL, FitzGerald LM, Liu E (2023). Predictive factors for treatment outcomes with intravitreal anti-vascular endothelial growth factor injections in diabetic macular edema in clinical practice. Int J Retina Vitreous.

[28] Usui-Ouchi A, Tamaki A, Sakanishi Y, Tamaki K, Mashimo K, Sakuma T, Ebihara N (2021). Factors affecting a short-term response to anti-VEGF therapy in diabetic macular edema. Life (Basel).

[29] Kakihara S, AbdelSalam M, Zhuang K, Fawzi AA (2025). Epiretinal membrane is associated with diabetic retinopathy severity and cumulative anti-VEGF injections. Ophthalmol Sci.

